# Liquid Biopsy in Pancreatic Ductal Adenocarcinoma: A Review of Methods and Applications

**DOI:** 10.3390/ijms252011013

**Published:** 2024-10-13

**Authors:** Genia Dubrovsky, Alison Ross, Pooya Jalali, Michael Lotze

**Affiliations:** 1 Department of Surgery, University of Pittsburgh, Pittsburgh, PA 15213, USA; dubrovskyge@upmc.edu (G.D.); alr264@pitt.edu (A.R.); 2 Pittsburgh VA Medical Center, Pittsburgh, PA 15240, USA; 3 Basic and Molecular Epidemiology of Gastrointestinal Disorders Research Centre, Research Institute for Gastroenterology and Liver Diseases, Shahid Beheshti University of Medical Sciences, Tehran 1983969411, Iran; 4 Departments of Surgery, Immunology, and Bioengineering, University of Pittsburgh, Pittsburgh, PA 15213, USA

**Keywords:** pancreatic cancer, liquid biopsy, ctDNA, exosomes

## Abstract

Pancreatic ductal adenocarcinoma (PDAC) remains a malignancy with one of the highest mortality rates. One limitation in the diagnosis and treatment of PDAC is the lack of an early and universal biomarker. Extensive research performed recently to develop new assays which could fit this role is available. In this review, we will discuss the current landscape of liquid biopsy in patients with PDAC. Specifically, we will review the various methods of liquid biopsy, focusing on circulating tumor DNA (ctDNA) and exosomes and future opportunities for improvement using artificial intelligence or machine learning to analyze results from a multi-omic approach. We will also consider applications which have been evaluated, including the utility of liquid biopsy for screening and staging patients at diagnosis as well as before and after surgery. We will also examine the potential for liquid biopsy to monitor patient treatment response in the setting of clinical trial development.

## 1. Introduction

Despite significant advances over the last two decades in the medical and surgical care of patients with pancreatic ductal adenocarcinoma (PDAC), the morbidity and mortality rates remain unacceptably high. The 5 year survival rate has increased to approximately 11%, remaining one of the lowest rates for any malignancy [1]. This is in part attributable to late disease presentation, with over 50% of patients already metastatic at the time of diagnosis [2,3]. Unfortunately, patients frequently have vague or non-specific symptoms which can delay diagnosis, and there is no approved screening test for the general population [3]. Typical diagnostic tools include cross-sectional imaging with CT or MRI, as well as endoscopic ultrasound and biopsies. However, these are limited by being costly and invasive, potentially leading to further procedures and surgeries which may be unnecessary [4]. PDAC is also a particularly aggressive cancer biologically, with early metastases being a hallmark of the disease [5]. At the same time, the incidence of pancreatic cancer is rising in both men and women and is predicted to become the second leading cause of cancer death by 2030–2040 [6,7]. Now more than ever, improved diagnostic and therapeutic strategies are needed to tackle pancreatic cancer.

One area being actively pursued is the development of novel biomarkers. Historically, CA 19-9 has been used most frequently to help in the diagnosis and treatment of PDAC [8,9,10]. It can predict patient survival, post-operative outcomes, and treatment response in patients with metastatic disease [8,9,10,11,12]. However, certain patients do not secrete CA 19-9 at all, and up to 1/3 of patients can have normal levels of it [13,14]. Furthermore, CA 19-9 lacks specificity to PDAC, as benign conditions and tumors other than PDAC can also have significantly elevated levels [15]. As a result, new noninvasive tests with improved sensitivity and specificity have been sought. Liquid biopsy in particular has garnered significant excitement in the field [16,17,18,19,20,21,22,23,24]. Liquid biopsy refers to analyzing a biofluid such as blood, saliva, or urine to obtain information about a patient’s tumor. By definition, these methods of diagnosis are less invasive than tissue biopsies, and the hope is that improved genetic methodologies and novel markers may provide optimal accuracy. In multiple other cancers such as melanoma, urothelial cancer, and colon cancer, liquid biopsy is showing promise as a predictive biomarker and even as one that can guide adjuvant treatment decisions [25,26,27,28]. In this review, we will focus on liquid biopsies which use cell free DNA (cfDNA) and exosomes to improve the care of patients with PDAC at different stages in the disease’s course. This review will prioritize publications from the last 15 years which discuss widely used methodologies, those assays which have been tested for clinical relevance, as well as those studies which were published more recently.

## 2. Methods of Liquid Biopsy in PDAC

**Figure 1:**
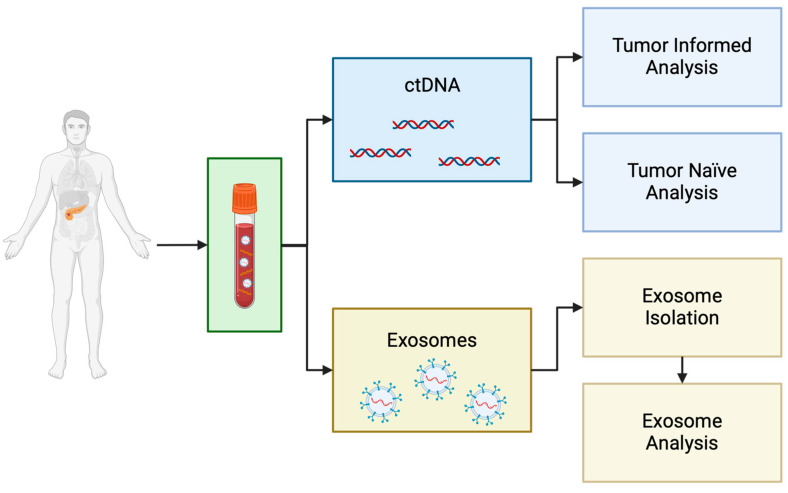
Liquid biopsy flow chart depicting potential analytes and progression of capture and evaluation. Created in BioRender by Ross, A. (2024) (BioRender.com/g43t071, Access date: 10 April 2024).

This section will discuss some of the more common methods of analyzing peripheral blood for evidence of PDAC. Two common analytes used for liquid biopsy include circulating tumor DNA (ctDNA) and exosomes. There are various methods for capturing and analyzing these biomarkers, as outlined in Figure 1 and Table 1.

### 2.1. Methods of ctDNA Analysis

Circulating tumor DNA (ctDNA) is a form of cfDNA released from tumor cells through apoptosis and necrosis [60]. These are short fragments of DNA which are approximately 166 base pairs in length on average [61]. Significant research has been devoted to optimizing techniques of ctDNA capture and analysis, with a common goal of improving diagnostics for a wide variety of malignancies. Tumor-informed analysis and tumor-naïve analysis present two primary groups of ctDNA tumor screening techniques where the patient’s tumor-specific genetic variations are known or unknown, respectively. About 88% of all PDAC cases will have a KRAS mutation, typically at the G12/13 and Q61 loci [62,63]. Therefore, evaluation for mutant KRAS in cfDNA is frequently a component of both tumor-informed and tumor-naïve analysis.

The following sections describe these different analysis methods for ctDNA, with a focus on common methodologies and the reported sensitivities and specificities of the various assays. While liquid biopsy has the advantage of being noninvasive, the accuracy of these tests is of the utmost importance. The gold standard is typically cross-sectional imaging with tissue biopsy for confirmation, as well as complementary evidence from CA 19-9 levels. If liquid biopsy techniques do not meet or exceed the accuracy of these current diagnostic tools, then they will have no clinical role. As will be discussed in subsequent sections, the clinical decisions for treatment escalation and de-escalation can only be reliably made when false positives and negatives are minimized.

#### 2.1.1. Tumor-Informed Analysis

In tumor-informed sequencing, cfDNA is screened for patient-specific tumor mutations or copy number alterations which have been identified by either sequencing the primary tumor or a tumor metastasis. Several techniques can be used to screen cfDNA, including qPCR, ddPCR, BEAMing, TAm-seq, and Safe-SeqS [29,30,31,32,33,34,35,36,37].

PCR has a longstanding history with targeted DNA sequencing. Two commonly used methods for ctDNA analysis are qPCR and droplet digital PCR (ddPCR), where ddPCR allows for partitioning tens of thousands of independent PCR reactions in a single test tube and thus has demonstrated improved precision compared with qPCR. This is particularly useful in detecting DNA targets present in a low abundance [29,30]. BEAMing and CAPP-seq are more sensitive than standard PCR as they combine emulsion PCR with flow cytometry and selector libraries, respectively, thus enabling the detection of smaller amounts of ctDNA [31,32,33,34]. BEAMing incorporates six major steps (coupling samples with beads, preparing microemulsions, PCR amplification, magnetic capture of beads, sequencing, and flow cytometry) to obtain a limit of detection near 0.01% [31,32]. CAPP-seq uses libraries of commonly mutated tumor genes to then perform deep sequencing of a patient’s tumor and ctDNA, achieving a limit of detection of about 0.0025% [33,34]. TAm-seq and Safe-SeqS are less sensitive than standard PCR (2% and 0.1%, respectively) but more sensitive than tumor-naïve sequencing. TAm-seq uses deep sequencing of common tumor mutations, while Safe-SeqS uses identifiers (barcodes or indexes) for specific genes of interest [35,36,37].

#### 2.1.2. Tumor-Naïve Analysis

While tumor-informed sequencing is useful for detecting lower concentrations of ctDNA and can be less costly, tumor-naïve sequencing is a useful tool when a primary tumor has not yet been identified or biopsied. For example, this may be useful in clinical scenarios where a recurrence is suspected but either radiographically occult or too small for a biopsy. Similarly, if a primary tumor cannot be biopsied, or the results are non-diagnostic, then a tumor-naïve assay may be ideal. This can also be an effective method for cancer screening, determining the mutational profile of a tumor, and identifying novel mutations which can develop over time for a known tumor. The three common methods are whole genome sequencing and whole exome sequencing (WGS and WES, respectively), methylation profiling, and fragmentomics [38,39,40,41,42,43]. WGS and WES sequence all the nucleic acids in the cfDNA sample and specifically the protein-coding cfDNA, respectively [38,39]. Both WGS and WES are often used to look for known PDAC signatures, such as KRAS mutations [64,65]. Methylation profiling uses an epigenetic approach to look for methylation signatures associated with tumors. This includes a genome-wide hypomethylation pattern with specific loci displaying hypermethylated signatures, particularly with regard to tumor suppressor genes [40,41]. While results with PDAC have been mixed, recent studies have shown promise, with the 10 marker set published by Vrba et al. having 100% sensitivity and 95% specificity in distinguishing metastatic PDAC from benign pancreatic cysts [66]. Similarly, Majumder et al. combined 13 methylation markers with CA 19-9 analysis to yield 92% sensitivity and a preset specificity of 97.5% for distinguishing all stages of PDAC from healthy controls [67]. Fragmentomics utilizes differences in cfDNA breakpoints and subsequent fragment lengths to trace the origin to tumor or non-tumor cells [42,43]. Cristiano et al. showed 65% sensitivity with 98% specificity in all stages of PDAC compared with healthy controls [42].

### 2.2. Methods of Exosome Analysis

Unlike ctDNA analysis, which studies cfDNA, exosome analysis investigates the contents on and within these extracellular vesicles (EVs), which can contain DNA, RNA, and proteins [68]. Exosomes are just one form of EVs, along with microvesicles and apoptotic bodies [44]. These forms of EVs differ in size and biogenesis. Exosomes tend to be the smallest, ranging in size from 40 to 120 nm, while microvesicles and apoptotic bodies range from 100 nm to 1 μm and 50 nm to 2 μm, respectively. Formation of these EVs also varies, with exosomes forming through the fusion of multivesicular bodies with the plasma membrane, microvesicles forming through plasma membrane budding, and apoptotic bodies forming through release from apoptotic cells [69]. It is believed that exosomes play a role in cell-to-cell communication, tumor progression, and immune system modulation [44]. During exosome synthesis, tumor cells deposit specific genes, proteins, and signal molecules which can reprogram target cells through mRNA silencing [68]. Since these exosomes have been released into the bloodstream (and other biofluids), they can be sampled using minimally invasive blood draws and can provide additional insights into the biology of the tumor from which they originated.

#### 2.2.1. Isolation

To analyze the exosomes, they must first be isolated and separated from other blood components and EVs. Current methods can be broken down into untargeted capture (size, density, etc.) and targeted capture (based on specific surface proteins) or a combination thereof. Prominent untargeted methods include ultracentrifugation, size-based filtration, and polyethylene glycol (PEG)- or lectin-induced precipitation, while targeted isolation techniques include immunoaffinity assays and immune-based microfluidics [44]. Table 2 summarizes the relative yields of some of these more prevalent methods [70].

The most commonly used isolation technique is differential ultrafiltration. However, due to the overlapping physical properties of many EVs, the yield is often impure [45]. Density gradient centrifugation utilizes similar density properties, but the incorporation of a gel, usually sucrose or iodoxinol, enables better removal of proteins, though other impurities remain [46]. Several filtration methods utilize size instead of density to isolate exosomes, including ultrafiltration, exosome isolation kits, sequential filtration, size exclusion chromatography, and flow field-flow fractionation (FIFFF) [44]. Another non-size-based, untargeted isolation method is PEG precipitation, during which PEG excludes water and causes exosomes and other particles to precipitate out [44,52]. Due to the lack of exosome specificity, PEG precipitation requires subsequent isolation steps [44,52]. Similarly, lectin-induced agglutination relies on lectin’s affinity to carbohydrate structures, including those located on the surface of exosomes, but like PEG, the nonspecific nature of this method requires pairing with other isolation options [44,53].

Targeted isolation methods, such as immunoaffinity assays and immune-based microfluidics, provide improved efficiency and specificity for select exosomes [44,46]. The primary method is magneto-immunoprecipitation, where magnetic beads are bound to antibodies which correspond to a protein of interest on the exosome and can be used to selectively elute exosomes expressing that protein [44,47]. Compared with ultracentrifugation, magneto-immunoprecipitation is quick and efficient, yielding a purer product which maintains more of its protein activity [44,47,54]. Similar to ELISA, immune-based microfluidics utilizes immobilized antibodies specific to a protein of interest on the surface of exosomes [44,55]. The benefits of using the microfluidic chip are that less of a sample is needed, and the assay can be run without previous isolation [44,55]. Several kits utilize this microfluidic technology, including ExoChip and ExoSearch Chip [44,55,56].

Signatures on the exosome membrane can differentiate tumor-derived cells from healthy cells of origin. For example, glypican-1 (GPC1) in PDAC initially showed some potential, with Melo et al. reporting 100% sensitivity and specificity in distinguishing PDAC patients from healthy patients or those with benign pancreatic disease [60,71]. However, it failed to differentiate exosomes derived from PDAC versus benign pancreatic cells in subsequent studies [72]. Lucien et al. found that GPC1 EV testing was only 26.67% sensitive and 87.50% specific when comparing PDAC and benign pancreatic disease [73]. Similarly, Frampton et al. showed that the GPC1 exosomes did not vary between patients with PDAC and benign pancreatic disease, although they did note a correlation between PDAC tumor size and GPC1 exosome levels [72]. In contrast, EphA2 shows potential as a PDAC marker, with Wei et al. reporting 82.5% sensitivity and 94.0% specificity when comparing early-stage PDAC to healthy patients and 84.4% sensitivity and 88.0% specificity when comparing PDAC to benign pancreatic disease [74,75]. These results improved further when combined with CA 19-9 levels [74].

#### 2.2.2. Analysis

Post isolation, further characterization analysis should be conducted, and this will be discussed in this section. Biochemical analysis of exosomes can characterize the DNA, RNA, and protein content. Many methods discussed in the ctDNA method section (PCR, WGS and WES, methylation, fragmentomics, etc.) are applicable to DNA and RNA analysis in exosomes after the nucleic acids have been isolated. Methods for studying the protein composition of exosomes include using antibodies to target proteins of interest via immunodetection, flow cytometry, and integrated immune isolation and proteomic analysis [44]. Thermophoretic profiling and mass spectrometry (MS) can also provide proteomic analysis [44]. For example, Marin et al. used MS-based proteomic analysis to identify differentially expressed proteins in the exosomes of patients with PDAC, intraductal papillary mucinous neoplasm (IPMN), and healthy controls [57]. Finally, some groups have also evaluated combined multi-omic approaches which use machine learning to find optimal combinations of exosomal nucleic acids and proteins for PDAC identification. Nakamura et al. used machine learning to find a panel of 13 cell-free and exosomal microRNAs to be used as a new biomarker for PDAC. When combined with CA 19-9, their assay had 93% sensitivity and 95% specificity for early-stage PDAC compared with healthy controls [58]. Similarly, Hinestrosa et al. used machine learning to select a panel of 13 EV-associated proteins which were able to detect early-stage PDAC from healthy controls with a sensitivity of 95.7% and specificity of 99.5% [59]. Both of these studies were limited by small or modest sample sizes and controls which used healthy patient samples rather than patients with benign pancreatic cysts or pancreatitis. Further studies will be needed to validate these results, but certainly they provide an exciting prospect of what may be possible in the future with the use of artificial intelligence (AI) and combinations of biomarkers.

AI is an up-and-coming analytical tool which has shown promise in the field of liquid biopsy. Eledkawy et al. demonstrated that their multi-method sequential analysis detects various cancers (including PDAC) with 99.45% accuracy and 99.95% AUC and classifies cancer types with 93.94% accuracy and a 97.81% AUC [76]. They used a combination of clinical features (age, sex, ethnicity, etc.), protein biomarkers (CA 19-9, CEA, etc.), and cfDNA mutations as the input for their model. They used AI to identify the top 10 predictive features to include in their final model. Other groups have also demonstrated >70% and >60% accuracy in detecting and classifying cancer, respectively, using platforms such as cancerSEEK, cancerAIDE, cancerEMC, and DEcancer individually [76,77,78,79,80]. While these studies demonstrate the promise of using AI in cancer diagnosis, this is a limited dataset with a focus on protein and DNA. Expanding the purview of these models to incorporate other pieces of information and over extended periods of time can create a more complete record of disease response and progression and thus may provide more insights.

## 3. Applications of Liquid Biopsy in PDAC

Liquid biopsy has been investigated at various disease stages to aid in different aspects of oncologic care. At the time of diagnosis, liquid biopsy has been used for screening purposes and to help predict patient outcomes from an early time point. Perioperatively, liquid biopsy can also be useful in predicting cancer recurrence and thus may aid in guiding treatment decisions for surgery and adjuvant care. Finally, liquid biopsy can assist in surveillance for cancer recurrence once patients have completed all treatments. With increasing frequency, liquid biopsy is also being used in clinical trial development. The following sections will discuss these different clinical applications for liquid biopsy. Table 3 highlights the key advantages and disadvantages associated with ctDNA and exosomes, which can be useful in determining the scenarios of use for each target. Some factors may have overlap between their advantages and disadvantages, depending on the planned application. For example, the half-life of ctDNA is typically to the order of hours, which may be useful in studying short-term changes [81]. However, this can also be a disadvantage in that ctDNA is less stable and can make sample processing more challenging.

### 3.1. Liquid Biopsy at Diagnosis

There are several studies which investigated the clinical applications of ctDNA assays at the time of diagnosis. Not surprisingly, liquid biopsy sensitivity correlates with the stage of the disease. The detection of ctDNA in patients with localized PDAC ranges from 10 to 48% [82,83,84,85]. The variability in detection rates may be attributable to several factors, including differences in patient populations and methodologies. For example, Sausen et al. showed a 43% detection rate in localized PDAC, but their population was primarily composed of node-positive PDAC or T3 tumors [84]. By contrast, the majority of the patients studied by Kirchweger et al. had T1 or T2 tumors which were clinically node-negative, and they reported a 10% detection rate [82]. Differences in ctDNA analysis can also affect the sensitivity of the assay. This includes both the actual assay used and differences in thresholding and prescribing cutoff values for assigning true positives. As previously discussed, ddPCR is a common assay used for ctDNA quantification, but the thresholds for true positives are not standardized. Some groups will use an MAF >0.1%, while others may set a cutoff of three positive droplets in a sample [82]. For example, Watanabe et al. showed that in localized PDAC, ctDNA detection rates were 39% when using a tumor-naïve approach with an MAF cutoff >0.065% and with at least two mutant allele copies detected. However, when they used a tumor-informed approach, they liberalized their cutoff values for known mutant alleles to any MAF and a mutant allele copy number of one. This increased the detection rate from 39% to 56% [86]. Regardless, even a sensitivity of 40–60% in the localized PDAC group is relatively modest, indicating that liquid biopsy may serve as a useful adjunct but is not yet ready to be a standalone universal biomarker for patients with resectable PDAC. This also highlights one limitation of trying to compare different study results. Each particular assay will have its own inherent limitations, and this can be further complicated when assays are applied to different patient populations. It is critical to recognize that any given liquid biopsy test is unique, unlike a standardized CA 19-9 level. Newer studies which use a multi-omic approach have reported much higher sensitivities (> 93%) in the early-stage population, but larger studies will be needed to validate these results, which are currently limited by small sample sizes [58,59].

In the metastatic population, ctDNA detection rates are generally much higher. Detection rates in this population have been reported to be 50–87% [82,83,85,87]. Furthermore, among patients with metastatic PDAC, those with greater numbers of liver metastases, with larger aggregate tumor sizes, and with the presence of peritoneal and lung metastases were also more likely to have elevated ctDNA levels [85,87]. Multiple studies have also shown the clinical significance of liquid biopsy at the time of diagnosis in PDAC before a patient starts any treatment. The results are best summarized in the meta-analysis by Guven et al., who reviewed 14 studies of ctDNA in localized PDAC and 22 studies of ctDNA in locally advanced or metastatic PDAC. For both patient populations, they noted that ctDNA positivity at the time of diagnosis was associated with significantly shorter recurrence-free survival, progression-free surivival, and overall survival periods [88]. This supports not only the utility of liquid biopsy in clinical care but, as will be discussed later, also highlights the value of liquid biopsy when allocating patients in clinical trials.

### 3.2. Liquid Biopsy Pre- and Postoperatively

Liquid biopsy has also shown utility peri-operatively as a biomarker which correlates with pathologic stage, molecular residual disease (MRD), and oncologic outcomes. In patients with non-metastatic PDAC, preoperative KRAS ctDNA detection by ddPCR correlated with lymph node positivity and the lymph node ratio [82]. The KRAS mutant allele fraction (MAF) was also higher in patients which were subsequently found to have occult metastatic disease upon surgical exploration [89]. However, only 41% of patients with occult metastatic disease had detectable ctDNA, and 14.6% of patients without metastasis also had detectable ctDNA [89]. Thus, this method of liquid biopsy cannot replace laparoscopy for accurate staging of patients. Intraoperative portal venous blood sampling has also been evaluated for its predictive value. Although the mutant KRAS copy value in portal venous blood was significantly higher than paired samples of peripheral blood, the KRAS mutation detection rate was not higher [90]. This is likely a consequence of more genomic cfDNA being released into the portal venous circulation during surgery. Nonetheless, positive detection of ctDNA in portal venous blood had the highest predictive value for short recurrence-free survival compared with blood taken from a peripheral stick, which is possibly related to the close anatomic proximity to the tumor [90]. Similar findings have been shown with EVs extracted from portal venous blood at the time of surgery. Specific types of exosomal microRNA were enriched in portal blood more than peripheral blood, and their presence correlated with worse PFS and OS results [91]. Ultimately, ctDNA in both preoperative blood samples and postoperative blood samples taken at any site correlated with patient recurrence-free survival and overall survival [90]. This has been shown in multiple studies on PDAC (Table 4) [84,90,92,93,94,95,96,97,98,99,100,101]. Depending on the study particulars (pathologic stage, use of neoadjuvant therapy, timing of liquid biopsy, and assay methodology), we see that about 15–50% of patients will have detectable ctDNA postoperatively.

In the largest retrospective study, Botta et al. reviewed tumor-informed ctDNA levels from 231 patients with stage 1–3 PDAC who all underwent curative-intent resection, in which 47% of the patients received neoadjuvant chemotherapy. MRD, as defined by ctDNA detection in the first 12 weeks following surgery, trended with the pathologic stage; 22.2% of patients with stage 1 PDAC had detectable ctDNA, compared with 19.4% of patients with stage 2 PDAC and 50% of patients with stage 3 PDAC [100]. Across all stages, patients with undetectable ctDNA postoperatively had a significantly improved disease-free survival rate compared with patients with detectable MRD (33.3 months versus 6.4 months). Interestingly, in this retrospective study, patients who had detectable MRD after surgery had similar disease-free survival regardless of whether or not they received additional adjuvant therapy. This was true both in the surgery-first group and in the neoadjuvant chemotherapy group. These results must be interpreted with caution though because this study was retrospective, and the sample sizes in these subgroups were quite small (only 12 patients with MRD after NAT and 17 patients with MRD after upfront surgery). There have been fewer clinical studies making use of exosomes to stratify patients perioperatively. Nonetheless, results have mirrored those seen with ctDNA [102,103,104]. For example, Nishiwada et al. identified six exosomal microRNAs analyzed in preoperative blood samples which could predict recurrence-free survival [103]. When combined with CA 19-9 levels, the AUC for the ROC curve improved to 0.84.

As a result of these findings, several strategies are being investigated to try to leverage the predictive power of ctDNA to improve treatment stratification of patients. For example, in the pre-op setting, Lee et al. raised the possibility that patients with positive liquid biopsy levels should try alternative systemic therapy options before proceeding to resection [94]. This is quite reasonable, since patients with an elevated pre-op ctDNA level have a worse OS. However, it is also important to note that 52% of patients with positive ctDNA preoperatively will clear the ctDNA level post-op [94]. These patients had a fair median OS of 15.9 months following surgery, particularly compared with patients with detectable ctDNA post-op who had a median OS of 10.6 months [94]. Thus although an elevated preoperative liquid biopsy could dictate escalation of systemic therapy, it should not necessarily preclude patients from consideration for resection. Similarly, Nitschke et al. suggested sampling blood from the portal vein for liquid biopsy at the time of diagnosis to help identify patients which would be treated with neoadjuvant chemotherapy, despite anatomic resectability of their tumors [90]. This treatment algorithm has yet to be interrogated in a clinical trial and thus has not yet been integrated into routine clinical practice. In the post-op setting, liquid biopsy positivity may help guide adjuvant treatment decisions, and this will be discussed further in the subsequent section.

### 3.3. Liquid Biopsy for Treatment Response and Clinical Trials

Patients with PDAC undergoing systemic treatment may also benefit from monitoring with liquid biopsy. Numerous studies have shown that longitudinal sampling for ctDNA and exosomes while patients undergo treatment with chemotherapy can help monitor response to treatment [85,105,106,107,108,109,110], where ctDNA and tumor exosomal DNA both showed a reduction in the MAF when patients responded to treatment and a rise in the MAF as the cancer became resistant to systemic therapy. Importantly, this fluctuation in MAF levels preceded radiographic evidence of progression by 50 days [85]. Whether or not this lead time can provide a meaningful opportunity for intervention is not yet known, but it may be useful as an early and objective endpoint in a clinical trial. In fact, multiple trials studying a wide range of malignancies have already started using liquid biopsy results as a trial endpoint [111,112,113,114,115]. In PDAC, ctDNA levels can also help predict responses to chemotherapy and new clinical trial drugs [116,117,118]. Furthermore, longitudinal liquid biopsies using tumor-naïve measures can also be useful for monitoring genetic variations which occur over time in a patient’s tumor [109]. In one case, this identified a novel mutation which induced resistance to the experimental drug being administered [119]. An important limitation to note, however, is that rates of liquid biopsy detection in the non-metastatic setting are still low for PDAC. This significantly limits the utility of liquid biopsy as a trial endpoint in early-stage disease. To overcome this challenge, it may be useful to enroll only patients who are positive at diagnosis and then monitor for biomarker clearance with treatment.

Another valuable application of liquid biopsy for clinical trials is at the time of enrollment. In order to appropriately enroll patients into trials with targeted therapies, the tumor mutational profile must be determined first. To optimize trial enrollment, Nakamura et al. compared patient enrollment following tumor genetic sequencing via tissue biopsy and via ctDNA liquid biopsy. They found that for advanced GI malignancies, genotyping via ctDNA was significantly faster than via tissue biopsy (11 days versus 33 days). They were also able to enroll a significantly higher proportion of patients (9.5% versus 4.1%) [120]. MAF as determined by liquid biopsy is also important to know at the time of trial enrollment so that patients can be appropriately stratified to treatment arms. The ctDNA level has been shown to be an important independent prognostic factor in progression-free survival for patients with metastatic PDAC and thus must be considered to ensure appropriate patient allocation in trials [121].

There are currently over 50 trials listed on ClinicalTrials.gov which are related to liquid biopsy in PDAC and enrolling patients [122,123]. These trials evaluate a variety of possible applications for liquid biopsy. Several trials will evaluate different liquid biopsy methods as a screening test for the general population. This would be a greatly welcomed resource as currently, there are no effective screening tests for PDAC in the general population, and as a result, the majority of patients present with stage 4 disease. There are also ongoing trials to try to determine if ctDNA positivity in patients with resectable PDAC warrants neodjuvant therapy prior to surgery. Multiple clinical trials will use liquid biopsy as a biomarker endpoint, and several studies are considering different biofluids for liquid biopsy, including peritoneal washings, cyst fluid, and stool studies. A French study, the PANLIPSY, will collect blood samples from 355 patients with and without PDAC to bank and analyze samples. Using AI, the group will evaluate numerous biomarkers, including ctDNA and exosomes, to identify and validate a liquid biopsy signature for PDAC [124]. Finally, several studies will evaluate whether MRD as measured by liquid biopsy after curative intent resection can help guide adjuvant treatment decisions. One such trial, the DYNAMIC-Pancreas trial from Australia, recently reported its preliminary results at ASCO 2024. This trial enrolled early-stage PDAC patients after upfront pancreatectomy and collected postoperative ctDNA samples. Among these patients, 44% with a negative ctDNA level had only 3 months of adjuvant chemothrapy at the discretion of the treating oncologist. By comparison, patients with positive ctDNA all had 6 months of adjuvant therapy. Long-term outcomes are still pending, but in line with prior studies, they did find that patients with a negative post-op ctDNA level had a longer RFS at 22 months, compared with 13 months in patients with a positive liquid biopsy [125]. Whether post-op ctDNA can guide treatment decisions in PDAC still has yet to be determined. In general, this multitude of clinical trials related to liquid biopsy in PDAC points to the excitement in the field and a hope that this new technology can significantly improve the care provided to our patients.

## 4. Conclusions

Liquid biopsy is a rapidly growing field within oncology in general but also as it pertains to pancreatic cancer specifically. Already, there are numerous new iterations of assays being developed with improving diagnostic accuracy. As discussed in this review, it is imperative for researchers and clinicians to be aware of the differences between assays and to recognize that results are not always comparable when different methodologies are used. Nonetheless, the clinical significance of both ctDNA and exosome analysis has been clearly demonstrated in numerous studies. Elevated blood ctDNA levels and tumor-derived exosomes portend poor outcomes at all stages and time points of a patient’s disease course. Importantly, liquid biopsy results can frequently detect disease progression several months prior to radiographic progression. Unfortunately, until the therapeutic options that are available to patients with PDAC improve, these results are not always actionable and are not always able to change the disease trajectory for a patient. This is a significant and somewhat unique limitation of the clinical utility of liquid biopsy in PDAC, and as a result, clinicians may wonder if there is any utility at all to these costly blood tests. As a point of rebuttal, we have already shown in this review that even now, liquid biopsy can greatly improve patient allocation to clinical trials and can expedite trial readout as an endpoint. Furthermore, one of the most exciting potentials for liquid biopsy is to identify an effective screening test for PDAC. While early studies have often focused on single gene mutations or single surface proteins for exosome capture, there is great promise in the ability to leverage AI to apply a multi-omic approach and to simultaneously evaluate a multitude of proteins and nucleic acids. This will not be possible without large collaborative studies and effective biobanking of specimens. Finally, treatment options for patients will undoubtably improve, and at that time, liquid biopsy will be instrumental in patient selection and guiding treatment escalation and de-escalation. As assays improve further, these novel biomarkers hold promise in helping with both the diagnosis and treatment of patients with PDAC.

## Figures and Tables

**Table 1:** Summary of commonly used liquid biopsy analytic methods using ctDNA and exosomes as substrates.

**ctDNA Analysis**	
**Tumor-Informed**	qPCR [29]
	ddPCR [29,30]
	BEAMing [31,32]
	CAPP-Seq [33,34]
	TAm-seq [35]
	Safe-SeqS [36,37]
**Tumor-Naïve**	WGS and WES [38,39]
	Methylation profiling [40,41]
	Fragmentomics [42,43]
**Exosome Analysis [44]**	
**Isolation**	Ultracentrifugation [45,46]
	Size-based separation [47,48,49,50,51]
	Precipitation [52,53]
	Immunoaffinity assay [46,47,54]
	Microfluidics [55,56]
***Analysis***	DNA characterization
	RNA characterization
	Proteomics [44,57]
	Combinatorial multi-omic approach with machine learning [58,59]

**Table 2:** Prominent exosome isolation methods and their relative yields.

Technique		Specificity	Recovery	Purity	Functionality of Exosome
**Ultracentrigugation**		moderate	moderate	high	moderate
**Size-based separation**	**Ultrafiltration**	low	high	low	moderate
	**Size exclusion chromatography**	moderate	high	high	high
**Precipitation**		low	high	low	moderate
**Immunoaffinity assay**		high	moderate	high	low
**Microfluidics**		high	low	high	low

Specificity refers to tumor-derived exosomes, recovery refers to total yield of exosomes, purity refers to the presence of additional contaminants, and functionality refers to whether exosomes can be studied in certain downstream assays [70].

**Table 3:** Advantages and disadvantages of ctDNA and exosomes as analytes in liquid biopsy [81].

Target	Advantages	Disadvantages
**ctDNA**	Less processing required	Less stable processing
	Identifying tumor status	Poor sensitivity when ctDNA concentration is low
	Quickly replaceable	Differentiating ctDNA from other cfDNA
		Less functional information
**Exosomes**	More stable	More processing required
	Information about multiple molecules (RNA, DNA, and protein)	Bias toward exosomal markers

**Table 4:** Results of studies evaluating outcomes of PDAC patients following curative intent surgical resection, stratified by postoperative ctDNA detection.

Study	Number of Patients	PDAC Stage	Neo-Adjuvant Therapy Given	Liquid Biopsy Method	Timing of Liquid Biopsy Post-Op	Detection Rate	ctDNA+ DFS	ctDNA- DFS	ctDNA+ OS	ctDNA- OS
Sausen et al., 2015 [84]	20	Stage 2 (resectable)	No	KRAS ddPCR	1–6.8 months	50.0%	9.9 months	Not reached *	-	-
Pietrasz et al., 2017 [92]	31	Unspecified	Unspecified	NGS	1–4 months	19.4%	4.6 months	17.6 months *	19.3 months	32.2 months **
Nakano et al., 2018 [93]	45	Stage 1–2$N0 22%	Yes (24.4%)	KRAS qPCR (with PNA clamping)	Prior to discharge	44.4%	~10 months	~24 months *	~19 months	~40 months **
Lee et al., 2019 [94]	35	Stage 1–2$T3 91%$N0 17%	No	KRAS SafeSeqS	4–8 weeks	37.1%	5.4 months	17.1 months *	10.6 months	Not reached **
Groot et al., 2019 [95]	41	Stage 1–3	Yes (41%)	KRAS ddPCR	Within 1 month	26.8%	5.0 months	15 months *	17 months	Not reached **
Jiang et al., 2020 [96]	27	Stage 1 (48%)$Stage 2 (33%)$Stage 4 (19%)	No	NGS	1 week	33.3%	~5 months	Not reached*	-	-
Yamaguchi et al., 2021 [97]	97	Stage 1 (4%)$Stage 2 (96%)$N0 36%	Yes (31%)	KRAS ddPCR	3 days	28%	6.9 months	19.2 months *	16.4 months	44.3 months **
Kitahata et al., 2022 [98]	27	Stage 1–2$T3 89%$N0 63%$(100% BR)	Yes (100%)	KRAS ddPCR	4–8 weeks	51.9%	11.1 months	12.3 months	23.8 months	Not reached **
Nitschke et al., 2023 [90]	37	Stage 1–3	Yes (40%)	KRAS ddPCR	1 week	24.3%	3.0 months	17.2 months *	-	-
Eckhoff et al., 2024 [99]	33	Stage 1–2$(resectable)	Yes (31%)	Signatera^TM^	Within 9 weeks	15.2%	3.2 months	9.8 months *	3.6 months	12.5 months **
Botta et al., 2024 [100]	100	Stage 1 (36%)$Stage 2 (36%)$Stage 3 (28%)	Yes (57%)	Signatera^TM^	2–12 weeks	29.0%	6.4 months	33.3 months *	-	-

DFS = median disease-free survival; OS = median overall survival; PNA = peptide nucleic acid. * Statistically significant difference in DFS. ** Statistically significant difference in OS.

## Data Availability

No new data were created or analyzed in this study. Data sharing is not applicable to this article.

## References

[1] Siegel R.L., Miller K.D., Fuchs H.E., Jemal A. (2022). Cancer statistics, 2022. CA Cancer J. Clin..

[2] House M.G., Gonen M., Jarnagin W.R., D’Angelica M., DeMatteo R.P., Fong Y., Brennan M.F., Allen P.J. (2007). Prognostic significance of pathologic nodal status in patients with resected pancreatic cancer. J. Gastrointest. Surg..

[3] Dbouk M., Katona B.W., Brand R.E., Chak A., Syngal S., Farrell J.J., Kastrinos F., Stoffel E.M., Blackford A.L., Rustgi A.K. (2022). The Multicenter Cancer of Pancreas Screening Study: Impact on Stage and Survival. J. Clin. Oncol..

[4] Wood L.D., Canto M.I., Jaffee E.M., Simeone D.M. (2022). Pancreatic Cancer: Pathogenesis, Screening, Diagnosis, and Treatment. Gastroenterology.

[5] Rhim A.D., Mirek E.T., Aiello N.M., Maitra A., Bailey J.M., McAllister F., Reichert M., Beatty G.L., Rustgi A.K., Vonderheide R.H. (2012). EMT and dissemination precede pancreatic tumor formation. Cell.

[6] Rahib L., Smith B.D., Aizenberg R., Rosenzweig A.B., Fleshman J.M., Matrisian L.M. (2014). Projecting cancer incidence and deaths to 2030: The unexpected burden of thyroid, liver, and pancreas cancers in the United States. Cancer Res..

[7] Rahib L., Wehner M.R., Matrisian L.M., Nead K.T. (2021). Estimated Projection of US Cancer Incidence and Death to 2040. JAMA Netw. Open.

[8] Boeck S., Stieber P., Holdenrieder S., Wilkowski R., Heinemann V. (2006). Prognostic and therapeutic significance of carbohydrate antigen 19-9 as tumor marker in patients with pancreatic cancer. Oncology.

[9] Boone B.A., Steve J., Zenati M.S., Hogg M.E., Singhi A.D., Bartlett D.L., Zureikat A.H., Bahary N., Zeh H.J. (2014). Serum CA 19-9 response to neoadjuvant therapy is associated with outcome in pancreatic adenocarcinoma. Ann. Surg. Oncol..

[10] Diaz C.L., Cinar P., Hwang J., Ko A.H., Tempero M.A. (2019). CA 19-9 Response: A Surrogate to Predict Survival in Patients With Metastatic Pancreatic Adenocarcinoma. Am. J. Clin. Oncol..

[11] Liu H., Zenati M.S., Rieser C.J., Al-Abbas A., Lee K.K., Singhi A.D., Bahary N., Hogg M.E., Zeh H.J., Zureikat A.H. (2020). CA19-9 Change During Neoadjuvant Therapy May Guide the Need for Additional Adjuvant Therapy Following Resected Pancreatic Cancer. Ann. Surg. Oncol..

[12] Reni M., Zanon S., Balzano G., Nobile S., Pircher C.C., Chiaravalli M., Passoni P., Arcidiacono P.G., Nicoletti R., Crippa S. (2017). Selecting patients for resection after primary chemotherapy for non-metastatic pancreatic adenocarcinoma. Ann. Oncol..

[13] Abdelrahman A.M., Goenka A.H., Alva-Ruiz R., Yonkus J.A., Leiting J.L., Graham R.P., Merrell K.W., Thiels C.A., Hallemeier C.L., Warner S.G. (2022). FDG-PET Predicts Neoadjuvant Therapy Response and Survival in Borderline Resectable/Locally Advanced Pancreatic Adenocarcinoma. J. Natl. Compr. Cancer Netw..

[14] Bergquist J.R., Puig C.A., Shubert C.R., Groeschl R.T., Habermann E.B., Kendrick M.L., Nagorney D.M., Smoot R.L., Farnell M.B., Truty M.J. (2016). Carbohydrate Antigen 19-9 Elevation in Anatomically Resectable, Early Stage Pancreatic Cancer Is Independently Associated with Decreased Overall Survival and an Indication for Neoadjuvant Therapy: A National Cancer Database Study. J. Am. Coll. Surg..

[15] Safi F., Schlosser W., Kolb G., Beger H.G. (1997). Diagnostic value of CA 19-9 in patients with pancreatic cancer and nonspecific gastrointestinal symptoms. J. Gastrointest. Surg..

[16] Ben-Ami R., Wang Q.L., Zhang J., Supplee J.G., Fahrmann J.F., Lehmann-Werman R., Brais L.K., Nowak J., Yuan C., Loftus M. (2024). Protein biomarkers and alternatively methylated cell-free DNA detect early stage pancreatic cancer. Gut.

[17] Garcia-Ortiz M.V., Cano-Ramirez P., Toledano-Fonseca M., Cano M.T., Inga-Saavedra E., Rodriguez-Alonso R.M., Guil-Luna S., Gomez-Espana M.A., Rodriguez-Ariza A., Aranda E. (2023). Circulating NPTX2 methylation as a non-invasive biomarker for prognosis and monitoring of metastatic pancreatic cancer. Clin. Epigenetics.

[18] Haan D., Bergamaschi A., Friedl V., Guler G.D., Ning Y., Reggiardo R., Kesling M., Collins M., Gibb B., Hazen K. (2023). Epigenomic Blood-Based Early Detection of Pancreatic Cancer Employing Cell-Free DNA. Clin. Gastroenterol. Hepatol..

[19] Hinestrosa J.P., Sears R.C., Dhani H., Lewis J.M., Schroeder G., Balcer H.I., Keith D., Sheppard B.C., Kurzrock R., Billings P.R. (2023). Development of a blood-based extracellular vesicle classifier for detection of early-stage pancreatic ductal adenocarcinoma. Commun. Med..

[20] Roy J.W., Wajnberg G., Ouellette A., Boucher J.E., Lacroix J., Chacko S., Ghosh A., Ouellette R.J., Lewis S.M. (2023). Small RNA sequencing analysis of peptide-affinity isolated plasma extracellular vesicles distinguishes pancreatic cancer patients from non-affected individuals. Sci. Rep..

[21] Ueda H., Takahashi H., Sakaniwa R., Kitamura T., Kobayashi S., Tomimaru Y., Kubo M., Sasaki K., Iwagami Y., Yamada D. (2024). Preoperative treatment response prediction for pancreatic cancer by multiple microRNAs in plasma exosomes: Optimization using machine learning and network analysis. Pancreatology.

[22] Uemura S., Kabe Y., Kitago M., Matsuda S., Abe Y., Hasegawa Y., Hori S., Tanaka M., Nakano Y., Sato Y. (2024). Prognosis prediction of PDAC via detection of O-glycan altered extracellular vesicles in perioperative sera. Cancer Sci..

[23] Wu Y., Seufert I., Al-Shaheri F.N., Kurilov R., Bauer A.S., Manoochehri M., Moskalev E.A., Brors B., Tjaden C., Giese N.A. (2023). DNA-methylation signature accurately differentiates pancreatic cancer from chronic pancreatitis in tissue and plasma. Gut.

[24] Zhao G., Jiang R., Shi Y., Gao S., Wang D., Li Z., Zhou Y., Sun J., Wu W., Peng J. (2024). Circulating cell-free DNA methylation-based multi-omics analysis allows early diagnosis of pancreatic ductal adenocarcinoma. Mol. Oncol..

[25] Lee J.H., Saw R.P., Thompson J.F., Lo S., Spillane A.J., Shannon K.F., Stretch J.R., Howle J., Menzies A.M., Carlino M.S. (2019). Pre-operative ctDNA predicts survival in high-risk stage III cutaneous melanoma patients. Ann. Oncol..

[26] Powles T., Assaf Z.J., Davarpanah N., Banchereau R., Szabados B.E., Yuen K.C., Grivas P., Hussain M., Oudard S., Gschwend J.E. (2021). ctDNA guiding adjuvant immunotherapy in urothelial carcinoma. Nature.

[27] Tie J., Cohen J.D., Lahouel K., Lo S.N., Wang Y., Kosmider S., Wong R., Shapiro J., Lee M., Harris S. (2022). Circulating Tumor DNA Analysis Guiding Adjuvant Therapy in Stage II Colon Cancer. N. Engl. J. Med..

[28] Tie J., Cohen J.D., Wang Y., Christie M., Simons K., Lee M., Wong R., Kosmider S., Ananda S., McKendrick J. (2019). Circulating Tumor DNA Analyses as Markers of Recurrence Risk and Benefit of Adjuvant Therapy for Stage III Colon Cancer. JAMA Oncol..

[29] Taylor S.C., Laperriere G., Germain H. (2017). Droplet Digital PCR versus qPCR for gene expression analysis with low abundant targets: From variable nonsense to publication quality data. Sci. Rep..

[30] Hindson B.J., Ness K.D., Masquelier D.A., Belgrader P., Heredia N.J., Makarewicz A.J., Bright I.J., Lucero M.Y., Hiddessen A.L., Legler T.C. (2011). High-throughput droplet digital PCR system for absolute quantitation of DNA copy number. Anal. Chem..

[31] Dressman D., Yan H., Traverso G., Kinzler K.W., Vogelstein B. (2003). Transforming single DNA molecules into fluorescent magnetic particles for detection and enumeration of genetic variations. Proc. Natl. Acad. Sci. USA.

[32] Diehl F., Schmidt K., Choti M.A., Romans K., Goodman S., Li M., Thornton K., Agrawal N., Sokoll L., Szabo S.A. (2008). Circulating mutant DNA to assess tumor dynamics. Nat. Med..

[33] Newman A.M., Bratman S.V., To J., Wynne J.F., Eclov N.C., Modlin L.A., Liu C.L., Neal J.W., Wakelee H.A., Merritt R.E. (2014). An ultrasensitive method for quantitating circulating tumor DNA with broad patient coverage. Nat. Med..

[34] Newman A.M., Lovejoy A.F., Klass D.M., Kurtz D.M., Chabon J.J., Scherer F., Stehr H., Liu C.L., Bratman S.V., Say C. (2016). Integrated digital error suppression for improved detection of circulating tumor DNA. Nat. Biotechnol..

[35] Forshew T., Murtaza M., Parkinson C., Gale D., Tsui D.W., Kaper F., Dawson S.J., Piskorz A.M., Jimenez-Linan M., Bentley D. (2012). Noninvasive identification and monitoring of cancer mutations by targeted deep sequencing of plasma DNA. Sci. Transl. Med..

[36] Kinde I., Wu J., Papadopoulos N., Kinzler K.W., Vogelstein B. (2011). Detection and quantification of rare mutations with massively parallel sequencing. Proc. Natl. Acad. Sci. USA.

[37] Fredebohm J., Mehnert D.H., Lober A.K., Holtrup F., van Rahden V., Angenendt P., Diehl F. (2016). Detection and Quantification of KIT Mutations in ctDNA by Plasma Safe-SeqS. Adv. Exp. Med. Biol..

[38] Norgaard M., Bjerre M.T., Fredsoe J., Vang S., Jensen J.B., De Laere B., Gronberg H., Borre M., Lindberg J., Sorensen K.D. (2023). Prognostic Value of Low-Pass Whole Genome Sequencing of Circulating Tumor DNA in Metastatic Castration-Resistant Prostate Cancer. Clin. Chem..

[39] Adalsteinsson V.A., Ha G., Freeman S.S., Choudhury A.D., Stover D.G., Parsons H.A., Gydush G., Reed S.C., Rotem D., Rhoades J. (2017). Scalable whole-exome sequencing of cell-free DNA reveals high concordance with metastatic tumors. Nat. Commun..

[40] Vaisvila R., Ponnaluri V.K.C., Sun Z., Langhorst B.W., Saleh L., Guan S., Dai N., Campbell M.A., Sexton B.S., Marks K. (2021). Enzymatic methyl sequencing detects DNA methylation at single-base resolution from picograms of DNA. Genome Res..

[41] Li Y., Fan Z., Meng Y., Liu S., Zhan H. (2023). Blood-based DNA methylation signatures in cancer: A systematic review. Biochim. Biophys. Acta Mol. Basis Dis..

[42] Cristiano S., Leal A., Phallen J., Fiksel J., Adleff V., Bruhm D.C., Jensen S.O., Medina J.E., Hruban C., White J.R. (2019). Genome-wide cell-free DNA fragmentation in patients with cancer. Nature.

[43] Esfahani M.S., Hamilton E.G., Mehrmohamadi M., Nabet B.Y., Alig S.K., King D.A., Steen C.B., Macaulay C.W., Schultz A., Nesselbush M.C. (2022). Inferring gene expression from cell-free DNA fragmentation profiles. Nat. Biotechnol..

[44] Doyle L.M., Wang M.Z. (2019). Overview of Extracellular Vesicles, Their Origin, Composition, Purpose, and Methods for Exosome Isolation and Analysis. Cells.

[45] Livshits M.A., Khomyakova E., Evtushenko E.G., Lazarev V.N., Kulemin N.A., Semina S.E., Generozov E.V., Govorun V.M. (2015). Isolation of exosomes by differential centrifugation: Theoretical analysis of a commonly used protocol. Sci. Rep..

[46] Tauro B.J., Greening D.W., Mathias R.A., Ji H., Mathivanan S., Scott A.M., Simpson R.J. (2012). Comparison of ultracentrifugation, density gradient separation, and immunoaffinity capture methods for isolating human colon cancer cell line LIM1863-derived exosomes. Methods.

[47] Li P., Kaslan M., Lee S.H., Yao J., Gao Z. (2017). Progress in Exosome Isolation Techniques. Theranostics.

[48] Gamez-Valero A., Monguio-Tortajada M., Carreras-Planella L., Franquesa M., Beyer K., Borras F.E. (2016). Size-Exclusion Chromatography-based isolation minimally alters Extracellular Vesicles’ characteristics compared to precipitating agents. Sci. Rep..

[49] Gheinani A.H., Vogeli M., Baumgartner U., Vassella E., Draeger A., Burkhard F.C., Monastyrskaya K. (2018). Improved isolation strategies to increase the yield and purity of human urinary exosomes for biomarker discovery. Sci. Rep..

[50] Vogel R., Coumans F.A., Maltesen R.G., Boing A.N., Bonnington K.E., Broekman M.L., Broom M.F., Buzas E.I., Christiansen G., Hajji N. (2016). A standardized method to determine the concentration of extracellular vesicles using tunable resistive pulse sensing. J. Extracell. Vesicles.

[51] Kang D., Oh S., Ahn S.M., Lee B.H., Moon M.H. (2008). Proteomic analysis of exosomes from human neural stem cells by flow field-flow fractionation and nanoflow liquid chromatography-tandem mass spectrometry. J. Proteome Res..

[52] Zeringer E., Barta T., Li M., Vlassov A.V. (2015). Strategies for isolation of exosomes. Cold Spring Harb. Protoc..

[53] Samsonov R., Shtam T., Burdakov V., Glotov A., Tsyrlina E., Berstein L., Nosov A., Evtushenko V., Filatov M., Malek A. (2016). Lectin-induced agglutination method of urinary exosomes isolation followed by mi-RNA analysis: Application for prostate cancer diagnostic. Prostate.

[54] Hong C.S., Muller L., Boyiadzis M., Whiteside T.L. (2014). Isolation and characterization of CD34+ blast-derived exosomes in acute myeloid leukemia. PLoS ONE.

[55] He M., Crow J., Roth M., Zeng Y., Godwin A.K. (2014). Integrated immunoisolation and protein analysis of circulating exosomes using microfluidic technology. Lab Chip.

[56] Kanwar S.S., Dunlay C.J., Simeone D.M., Nagrath S. (2014). Microfluidic device (ExoChip) for on-chip isolation, quantification and characterization of circulating exosomes. Lab Chip.

[57] Marin A.M., Batista M., Korte de Azevedo A.L., Bombardelli Gomig T.H., Soares Caldeira Brant R., Chammas R., Uno M., Dias Araujo D., Zanette D.L., Nobrega Aoki M. (2023). Screening of Exosome-Derived Proteins and Their Potential as Biomarkers in Diagnostic and Prognostic for Pancreatic Cancer. Int. J. Mol. Sci..

[58] Nakamura K., Zhu Z., Roy S., Jun E., Han H., Munoz R.M., Nishiwada S., Sharma G., Cridebring D., Zenhausern F. (2022). An Exosome-based Transcriptomic Signature for Noninvasive, Early Detection of Patients With Pancreatic Ductal Adenocarcinoma: A Multicenter Cohort Study. Gastroenterology.

[59] Hinestrosa J.P., Kurzrock R., Lewis J.M., Schork N.J., Schroeder G., Kamat A.M., Lowy A.M., Eskander R.N., Perrera O., Searson D. (2022). Early-stage multi-cancer detection using an extracellular vesicle protein-based blood test. Commun. Med..

[60] Hsu S.K., Jadhao M., Liao W.T., Chang W.T., Lin I.L., Chiu C.C. (2023). The Role of Exosomes in Pancreatic Ductal Adenocarcinoma Progression and Their Potential as Biomarkers. Cancers.

[61] Jiang P., Chan C.W., Chan K.C., Cheng S.H., Wong J., Wong V.W., Wong G.L., Chan S.L., Mok T.S., Chan H.L. (2015). Lengthening and shortening of plasma DNA in hepatocellular carcinoma patients. Proc. Natl. Acad. Sci. USA.

[62] Singhi A.D., George B., Greenbowe J.R., Chung J., Suh J., Maitra A., Klempner S.J., Hendifar A., Milind J.M., Golan T. (2019). Real-Time Targeted Genome Profile Analysis of Pancreatic Ductal Adenocarcinomas Identifies Genetic Alterations That Might Be Targeted With Existing Drugs or Used as Biomarkers. Gastroenterology.

[63] Luo J. (2021). KRAS mutation in pancreatic cancer. Semin. Oncol..

[64] Huerta M., Martin-Arana J., Gimeno-Valiente F., Carbonell-Asins J.A., Garcia-Mico B., Martinez-Castedo B., Robledo-Yague F., Camblor D.G., Fleitas T., Garcia Bartolome M. (2024). ctDNA whole exome sequencing in pancreatic ductal adenocarcinoma unveils organ-dependent metastatic mechanisms and identifies actionable alterations in fast progressing patients. Transl. Res..

[65] Wei T., Zhang J., Li J., Chen Q., Zhi X., Tao W., Ma J., Yang J., Lou Y., Ma T. (2020). Genome-wide profiling of circulating tumor DNA depicts landscape of copy number alterations in pancreatic cancer with liver metastasis. Mol. Oncol..

[66] Vrba L., Futscher B.W., Oshiro M., Watts G.S., Menashi E., Hu C., Hammad H., Pennington D.R., Golconda U., Gavini H. (2022). Liquid biopsy, using a novel DNA methylation signature, distinguishes pancreatic adenocarcinoma from benign pancreatic disease. Clin. Epigenetics.

[67] Majumder S., Taylor W.R., Foote P.H., Berger C.K., Wu C.W., Mahoney D.W., Bamlet W.R., Burger K.N., Postier N., de la Fuente J. (2021). High Detection Rates of Pancreatic Cancer Across Stages by Plasma Assay of Novel Methylated DNA Markers and CA19-9. Clin. Cancer Res..

[68] Melo S.A., Sugimoto H., O’Connell J.T., Kato N., Villanueva A., Vidal A., Qiu L., Vitkin E., Perelman L.T., Melo C.A. (2014). Cancer Exosomes Perform Cell-Independent MicroRNA Biogenesis and Promote Tumorigenesis. Cancer Cell.

[69] Zaborowski M.P., Balaj L., Breakefield X.O., Lai C.P. (2015). Extracellular Vesicles: Composition, Biological Relevance, and Methods of Study. Bioscience.

[70] Sidhom K., Obi P.O., Saleem A. (2020). A Review of Exosomal Isolation Methods: Is Size Exclusion Chromatography the Best Option?. Int. J. Mol. Sci..

[71] Melo S.A., Luecke L.B., Kahlert C., Fernandez A.F., Gammon S.T., Kaye J., LeBleu V.S., Mittendorf E.A., Weitz J., Rahbari N. (2015). Glypican-1 identifies cancer exosomes and detects early pancreatic cancer. Nature.

[72] Frampton A.E., Prado M.M., Lopez-Jimenez E., Fajardo-Puerta A.B., Jawad Z.A.R., Lawton P., Giovannetti E., Habib N.A., Castellano L., Stebbing J. (2018). Glypican-1 is enriched in circulating-exosomes in pancreatic cancer and correlates with tumor burden. Oncotarget.

[73] Lucien F., Lac V., Billadeau D.D., Borgida A., Gallinger S., Leong H.S. (2019). Glypican-1 and glycoprotein 2 bearing extracellular vesicles do not discern pancreatic cancer from benign pancreatic diseases. Oncotarget.

[74] Wei Q., Zhang J., Li Z., Wei L., Ren L. (2020). Serum Exo-EphA2 as a Potential Diagnostic Biomarker for Pancreatic Cancer. Pancreas.

[75] Liang K., Liu F., Fan J., Sun D., Liu C., Lyon C.J., Bernard D.W., Li Y., Yokoi K., Katz M.H. (2017). Nanoplasmonic Quantification of Tumor-derived Extracellular Vesicles in Plasma Microsamples for Diagnosis and Treatment Monitoring. Nat. Biomed. Eng..

[76] Eledkawy A., Hamza T., El-Metwally S. (2024). Precision cancer classification using liquid biopsy and advanced machine learning techniques. Sci. Rep..

[77] Cohen J.D., Li L., Wang Y., Thoburn C., Afsari B., Danilova L., Douville C., Javed A.A., Wong F., Mattox A. (2018). Detection and localization of surgically resectable cancers with a multi-analyte blood test. Science.

[78] Wong K.C., Chen J., Zhang J., Lin J., Yan S., Zhang S., Li X., Liang C., Peng C., Lin Q. (2019). Early Cancer Detection from Multianalyte Blood Test Results. iScience.

[79] Rahaman S., Li X., Yu J., Wong K.C. (2021). CancerEMC: Frontline non-invasive cancer screening from circulating protein biomarkers and mutations in cell-free DNA. Bioinformatics.

[80] Halner A., Hankey L., Liang Z., Pozzetti F., Szulc D., Mi E., Liu G., Kessler B.M., Syed J., Liu P.J. (2023). DEcancer: Machine learning framework tailored to liquid biopsy based cancer detection and biomarker signature selection. iScience.

[81] Jia S., Zhang R., Li Z., Li J. (2017). Clinical and biological significance of circulating tumor cells, circulating tumor DNA, and exosomes as biomarkers in colorectal cancer. Oncotarget.

[82] Kirchweger P., Kupferthaler A., Burghofer J., Webersinke G., Jukic E., Schwendinger S., Weitzendorfer M., Petzer A., Fugger R., Rumpold H. (2022). Circulating tumor DNA correlates with tumor burden and predicts outcome in pancreatic cancer irrespective of tumor stage. Eur. J. Surg. Oncol..

[83] Bettegowda C., Sausen M., Leary R.J., Kinde I., Wang Y., Agrawal N., Bartlett B.R., Wang H., Luber B., Alani R.M. (2014). Detection of circulating tumor DNA in early- and late-stage human malignancies. Sci. Transl. Med..

[84] Sausen M., Phallen J., Adleff V., Jones S., Leary R.J., Barrett M.T., Anagnostou V., Parpart-Li S., Murphy D., Kay Li Q. (2015). Clinical implications of genomic alterations in the tumour and circulation of pancreatic cancer patients. Nat. Commun..

[85] Bernard V., Kim D.U., San Lucas F.A., Castillo J., Allenson K., Mulu F.C., Stephens B.M., Huang J., Semaan A., Guerrero P.A. (2019). Circulating Nucleic Acids Are Associated With Outcomes of Patients With Pancreatic Cancer. Gastroenterology.

[86] Watanabe K., Nakamura T., Kimura Y., Motoya M., Kojima S., Kuraya T., Murakami T., Kaneko T., Shinohara Y., Kitayama Y. (2022). Tumor-Informed Approach Improved ctDNA Detection Rate in Resected Pancreatic Cancer. Int. J. Mol. Sci..

[87] Uesato Y., Sasahira N., Ozaka M., Sasaki T., Takatsuki M., Zembutsu H. (2020). Evaluation of circulating tumor DNA as a biomarker in pancreatic cancer with liver metastasis. PLoS ONE.

[88] Guven D.C., Sahin T.K., Yildirim H.C., Aktepe O.H., Dizdar O., Yalcin S. (2021). A systematic review and meta-analysis of the association between circulating tumor DNA (ctDNA) and prognosis in pancreatic cancer. Crit. Rev. Oncol. Hematol..

[89] Hata T., Mizuma M., Iseki M., Takadate T., Ishida M., Nakagawa K., Hayashi H., Morikawa T., Motoi F., Unno M. (2021). Circulating tumor DNA as a predictive marker for occult metastases in pancreatic cancer patients with radiographically non-metastatic disease. J. Hepatobiliary Pancreat. Sci..

[90] Nitschke C., Markmann B., Walter P., Badbaran A., Tolle M., Kropidlowski J., Belloum Y., Goetz M.R., Bardenhagen J., Stern L. (2023). Peripheral and Portal Venous KRAS ctDNA Detection as Independent Prognostic Markers of Early Tumor Recurrence in Pancreatic Ductal Adenocarcinoma. Clin. Chem..

[91] Kawamura S., Iinuma H., Wada K., Takahashi K., Minezaki S., Kainuma M., Shibuya M., Miura F., Sano K. (2019). Exosome-encapsulated microRNA-4525, microRNA-451a and microRNA-21 in portal vein blood is a high-sensitive liquid biomarker for the selection of high-risk pancreatic ductal adenocarcinoma patients. J. Hepatobiliary Pancreat. Sci..

[92] Pietrasz D., Pecuchet N., Garlan F., Didelot A., Dubreuil O., Doat S., Imbert-Bismut F., Karoui M., Vaillant J.C., Taly V. (2017). Plasma Circulating Tumor DNA in Pancreatic Cancer Patients Is a Prognostic Marker. Clin. Cancer Res..

[93] Nakano Y., Kitago M., Matsuda S., Nakamura Y., Fujita Y., Imai S., Shinoda M., Yagi H., Abe Y., Hibi T. (2018). KRAS mutations in cell-free DNA from preoperative and postoperative sera as a pancreatic cancer marker: A retrospective study. Br. J. Cancer.

[94] Lee B., Lipton L., Cohen J., Tie J., Javed A.A., Li L., Goldstein D., Burge M., Cooray P., Nagrial A. (2019). Circulating tumor DNA as a potential marker of adjuvant chemotherapy benefit following surgery for localized pancreatic cancer. Ann. Oncol..

[95] Groot V.P., Mosier S., Javed A.A., Teinor J.A., Gemenetzis G., Ding D., Haley L.M., Yu J., Burkhart R.A., Hasanain A. (2019). Circulating Tumor DNA as a Clinical Test in Resected Pancreatic Cancer. Clin. Cancer Res..

[96] Jiang J., Ye S., Xu Y., Chang L., Hu X., Ru G., Guo Y., Yi X., Yang L., Huang D. (2020). Circulating Tumor DNA as a Potential Marker to Detect Minimal Residual Disease and Predict Recurrence in Pancreatic Cancer. Front. Oncol..

[97] Yamaguchi T., Uemura K., Murakami Y., Kondo N., Nakagawa N., Okada K., Seo S., Hiyama E., Takahashi S., Sueda T. (2021). Clinical Implications of Pre- and Postoperative Circulating Tumor DNA in Patients with Resected Pancreatic Ductal Adenocarcinoma. Ann. Surg. Oncol..

[98] Kitahata Y., Kawai M., Hirono S., Okada K.I., Miyazawa M., Motobayashi H., Ueno M., Hayami S., Miyamoto A., Yamaue H. (2022). Circulating Tumor DNA as a Potential Prognostic Marker in Patients with Borderline-Resectable Pancreatic Cancer Undergoing Neoadjuvant Chemotherapy Followed by Pancreatectomy. Ann. Surg. Oncol..

[99] Eckhoff A.M., Kanu E., Fletcher A., Bao M., Aushev V.N., Spickard E., Nussbaum D.P., Allen P.J. (2024). Initial Report: Personalized Circulating Tumor DNA and Survival in Patients with Resectable Pancreatic Cancer. Ann. Surg. Oncol..

[100] Botta G.P., Abdelrahim M., Drengler R.L., Aushev V.N., Esmail A., Laliotis G., Brewer C.M., George G.V., Abbate S.M., Chandana S.R. (2024). Association of personalized and tumor-informed ctDNA with patient survival outcomes in pancreatic adenocarcinoma. Oncologist.

[101] Li S., Zhang G., Li X., Li X., Chen X., Xu Y., Ren H. (2021). Role of the preoperative circulating tumor DNA KRAS mutation in patients with resectable pancreatic cancer. Pharmacogenomics.

[102] Huang L., Yuan X., Zhao L., Han Q., Yan H., Yuan J., Guan S., Xu X., Dai G., Wang J. (2023). Gene signature developed for predicting early relapse and survival in early-stage pancreatic cancer. BJS Open.

[103] Nishiwada S., Cui Y., Sho M., Jun E., Akahori T., Nakamura K., Sonohara F., Yamada S., Fujii T., Han I.W. (2022). Transcriptomic Profiling Identifies an Exosomal microRNA Signature for Predicting Recurrence Following Surgery in Patients With Pancreatic Ductal Adenocarcinoma. Ann. Surg..

[104] Takahasi K., Iinuma H., Wada K., Minezaki S., Kawamura S., Kainuma M., Ikeda Y., Shibuya M., Miura F., Sano K. (2018). Usefulness of exosome-encapsulated microRNA-451a as a minimally invasive biomarker for prediction of recurrence and prognosis in pancreatic ductal adenocarcinoma. J. Hepatobiliary Pancreat. Sci..

[105] Tjensvoll K., Lapin M., Buhl T., Oltedal S., Steen-Ottosen Berry K., Gilje B., Soreide J.A., Javle M., Nordgard O., Smaaland R. (2016). Clinical relevance of circulating KRAS mutated DNA in plasma from patients with advanced pancreatic cancer. Mol. Oncol..

[106] Perets R., Greenberg O., Shentzer T., Semenisty V., Epelbaum R., Bick T., Sarji S., Ben-Izhak O., Sabo E., Hershkovitz D. (2018). Mutant KRAS Circulating Tumor DNA Is an Accurate Tool for Pancreatic Cancer Monitoring. Oncologist.

[107] Park G., Park J.K., Son D.S., Shin S.H., Kim Y.J., Jeon H.J., Lee J., Park W.Y., Lee K.H., Park D. (2018). Utility of targeted deep sequencing for detecting circulating tumor DNA in pancreatic cancer patients. Sci. Rep..

[108] Wei T., Zhang Q., Li X., Su W., Li G., Ma T., Gao S., Lou J., Que R., Zheng L. (2019). Monitoring Tumor Burden in Response to FOLFIRINOX Chemotherapy Via Profiling Circulating Cell-Free DNA in Pancreatic Cancer. Mol. Cancer Ther..

[109] Sugimori M., Sugimori K., Tsuchiya H., Suzuki Y., Tsuyuki S., Kaneta Y., Hirotani A., Sanga K., Tozuka Y., Komiyama S. (2020). Quantitative monitoring of circulating tumor DNA in patients with advanced pancreatic cancer undergoing chemotherapy. Cancer Sci..

[110] Sivapalan L., Thorn G.J., Gadaleta E., Kocher H.M., Ross-Adams H., Chelala C. (2022). Longitudinal profiling of circulating tumour DNA for tracking tumour dynamics in pancreatic cancer. BMC Cancer.

[111] Ren S., Chen J., Xu X., Jiang T., Cheng Y., Chen G., Pan Y., Fang Y., Wang Q., Huang Y. (2022). Camrelizumab Plus Carboplatin and Paclitaxel as First-Line Treatment for Advanced Squamous NSCLC (CameL-Sq): A Phase 3 Trial. J. Thorac. Oncol..

[112] Provencio M., Serna-Blasco R., Nadal E., Insa A., Garcia-Campelo M.R., Casal Rubio J., Domine M., Majem M., Rodriguez-Abreu D., Martinez-Marti A. (2022). Overall Survival and Biomarker Analysis of Neoadjuvant Nivolumab Plus Chemotherapy in Operable Stage IIIA Non-Small-Cell Lung Cancer (NADIM phase II trial). J. Clin. Oncol..

[113] Carvajal R.D., Butler M.O., Shoushtari A.N., Hassel J.C., Ikeguchi A., Hernandez-Aya L., Nathan P., Hamid O., Piulats J.M., Rioth M. (2022). Clinical and molecular response to tebentafusp in previously treated patients with metastatic uveal melanoma: A phase 2 trial. Nat. Med..

[114] Maron S.B., Chatila W., Walch H., Chou J.F., Ceglia N., Ptashkin R., Do R.K.G., Paroder V., Pandit-Taskar N., Lewis J.S. (2023). Determinants of Survival with Combined HER2 and PD-1 Blockade in Metastatic Esophagogastric Cancer. Clin. Cancer Res..

[115] Anagnostou V., Ho C., Nicholas G., Juergens R.A., Sacher A., Fung A.S., Wheatley-Price P., Laurie S.A., Levy B., Brahmer J.R. (2023). ctDNA response after pembrolizumab in non-small cell lung cancer: Phase 2 adaptive trial results. Nat. Med..

[116] Bachet J.B., Blons H., Hammel P., Hariry I.E., Portales F., Mineur L., Metges J.P., Mulot C., Bourreau C., Cain J. (2020). Circulating Tumor DNA is Prognostic and Potentially Predictive of Eryaspase Efficacy in Second-line in Patients with Advanced Pancreatic Adenocarcinoma. Clin. Cancer Res..

[117] Evrard C., Ingrand P., Rochelle T., Martel M., Tachon G., Flores N., Randrian V., Ferru A., Haineaux P.A., Goujon J.M. (2023). Circulating tumor DNA in unresectable pancreatic cancer is a strong predictor of first-line treatment efficacy: The KRASCIPANC prospective study. Dig. Liver Dis..

[118] Kubendran S., Boland J.L., Jurdi A.A., Ween A., Baker G., Ma H., Baffa R., Li Z., Botta G.P. (2023). Circulating tumor DNA and association with CAR-T cell therapy response in gastric and pancreatic cancer patients. J. Clin. Oncol..

[119] Aung K.L., McWhirter E., Welch S., Wang L., Lovell S., Stayner L.A., Ali S., Malpage A., Makepeace B., Ramachandran M. (2022). A phase II trial of GSK2256098 and trametinib in patients with advanced pancreatic ductal adenocarcinoma. J. Gastrointest. Oncol..

[120] Nakamura Y., Taniguchi H., Ikeda M., Bando H., Kato K., Morizane C., Esaki T., Komatsu Y., Kawamoto Y., Takahashi N. (2020). Clinical utility of circulating tumor DNA sequencing in advanced gastrointestinal cancer: SCRUM-Japan GI-SCREEN and GOZILA studies. Nat. Med..

[121] Pietrasz D., Wang-Renault S., Taieb J., Dahan L., Postel M., Durand-Labrunie J., Le Malicot K., Mulot C., Rinaldi Y., Phelip J.M. (2022). Prognostic value of circulating tumour DNA in metastatic pancreatic cancer patients: Post-hoc analyses of two clinical trials. Br. J. Cancer.

[122] ClinicalTrials.gov. Search Results for Pancreatic Cancer and ctDNA. https://clinicaltrials.gov/search?cond=Pancreatic%20Cancer&term=ctdna&limit=100.&viewType=Card.

[123] ClinicalTrials.gov. Search Results for Pancreatic Cancer and Exosomes. https://clinicaltrials.gov/search?cond=Pancreatic%20Cancer&term=exosomes&limit=100.

[124] Bardol T., Dujon A.M., Taly V., Dunyach-Remy C., Lavigne J.P., Costa-Silva B., Kurma K., Eslami S.Z., Cayrefourcq L., Canivet C. (2024). Early detection of pancreatic cancer by liquid biopsy “PANLIPSY”: A french nation-wide study project. BMC Cancer.

[125] Lee B., Tie J., Wang Y., Cohen J.D., Shapiro J.D., Wong R., Aghmesheh M., Kiberu A.D., Francesconi A., Burge M.E. (2024). The potential role of serial circulating tumor DNA (ctDNA) testing after upfront surgery to guide adjuvant chemotherapy for early stage pancreatic cancer: The AGITG DYNAMIC-Pancreas trial. J. Clin. Oncol..

